# The impact of Boric Acid tubes on quantitative urinary bacterial cultures in hospitalized patients

**DOI:** 10.1007/s10096-024-04874-z

**Published:** 2024-06-25

**Authors:** Debby Ben-David, Yael Cohen, Iris Zohar, Yasmin Maor, Orna Schwartz

**Affiliations:** 1 Infection Control Unit, Wolfson Medical Center, Ha-Lokhamim St 62, Holon, 5822012 Israel; 2 Faculty of Medicine, Tel Aviv University, Tel Aviv, Israel; 3 Infectious Disease Unit, Wolfson Medical Center, Holon, Israel; 4 Microbiology Laboratory, Wolfson Medical Center, Holon, Israel

**Keywords:** Boric acid, Urine cultures

## Abstract

**Introduction:**

The accuracy of urine culture results can be affected by pre-analytical factors such as transport delays and storage conditions. The objectives of this study were to analyze urine collection practices and assess the impact of introducing boric acid tubes for urine collection on quantitative urinary bacterial cultures of hospitalized patients in medical wards.

**Methods:**

A quasi-experimental pre-post study conducted in an acute care facility. In the pre-intervention phase (2020–2021), urine samples were transported without preservatives at room temperature. In 2022 (post-intervention), we transitioned to boric acid transport tubes, evaluating its effect on significant bacterial growth (≥ 105 CFU/ml). Bivariate and multivariate analyses identified predictors of culture positivity.

**Results:**

Throughout the duration of the study, a total of 12,660 urine cultures were analyzed. Date and time documentation was complete for 38.3% of specimens. Culture positivity was higher with longer processing times: positivity was 21.3% (220/1034) when specimens were processed within 4 h, 28.4% (955/3364) when processed in 4–24 h, and 32.9% (137/417) when processed after 24 h (*p* < 0.0001). For 4-24-hour processing, positivity decreased from 30.4% (704/2317) pre-intervention to 24.0% (251/1047) post-intervention (*p* < 0.001), with no significant changes in < 4 or ≥ 24-hour specimens. Stratified analysis by processing time revealed that the intervention was associated with reduced positivity only in cultures processed within 4–24 h (OR 0.80, 95% CI 0.67–0.94; *p* = 0.008).

**Conclusion:**

The introduction of boric acid transport tubes predominantly influenced cultures transported within a 4–24-hour window. This presents an opportunity to improve urine tract infection diagnostic practices in healthcare settings.

## Introduction

Urinary tract infections (UTIs) are among the most prevalent infections in both community and hospital settings. They are linked to substantial morbidity, antibiotic usage, and significant financial expenses [1, 2]. Accurate diagnosis of patients who are exhibiting symptoms of UTI is crucial for applying appropriate antibiotic stewardship, as it helps to prevent both underutilization and overutilization of antimicrobial treatment [3].

A UTI is defined as the growth of bacteria in a urine culture at a concentration above 10 [5] colony forming units per milliliter (CFU/ml) in combination with local or systemic symptoms [4]. Hence, proper collection of urine for culture should have several goals: identify a causative pathogen if present, preserve the organism at a colony count that reflects the patient’s clinical condition at the time of collection, and avoid introduction of a contaminant into the specimen. Overgrowth of a pathogen or mistaking a contaminant for a pathogen may lead to unnecessary antibiotic treatment. The accuracy of urine culture results is susceptible to several pre-analytical factors, such as the method of collection, delay in transport, and storage conditions [5] The gold standard for diagnosis of a UTI is identification of a pathogen in a freshly collected specimen of urine. Previous studies have evaluated the impact of delays in transporting or processing of urine specimens [6, 7] Urine held at room temperature for more than 4 h shows overgrowth of both clinically significant and contaminating microorganisms. This may lead to false-positive results and misdiagnosis of urinary tract infections. Prior research has shown that transport tubes containing boric acid as a preservative effectively inhibit bacterial overgrowth, thus reducing the potential for false-positive results [8–10]. However, due to the relatively low number of samples tested, the strength of the evidence was considered low, and the routine use of boric acid tubes for urine sample collection was not recommended [5]. The objectives of this study were to analyze urine collection practices and assess the impact of introducing boric acid tubes for urine collection on quantitative urinary bacterial cultures of hospitalized patients in medical wards.

## Methods

The study was conducted at the Wolfson Medical Center, a 720-bed secondary-care teaching hospital situated in central Israel. The facility houses six medical wards with a median patient age of 78 years. Our research was a quasi-experimental pre-post investigation involving all urine cultures collected from patients aged 18 years and older who were hospitalized in medical wards.

During the pre-intervention phase (January 2020-December 2021), Urine cultures were collected in sterile, preservative-free containers, stored at room temperature, and transported to the microbiological laboratory for processing. During the intervention phase (January 2022-December 2022), urine specimens were collected using boric acid tubes (BD Vacutainer® C&S Boric Acid) and transported via the pneumatic transport system.

### Laboratory methods

Identical laboratory procedures were applied during both phases. Urine samples were cultured semiquantitatively on Blood Agar / CHROMagar Orientation plates (Hy-Laboratories Ltd, Rehovot, Israel) using the calibrated loop technique and incubated overnight at 37 C°. Each urine culture result was categorized as negative, mixed flora, <100,000 CFU/ml, or ≥ 100,000 CFU/ml (significant bacterial growth). We employed the definition of UTI used for surveillance purposes by the US. National Healthcare Safety Network [11] as well as in several recent studies on. diagnostic stewardship [12–14].

### Data Collection and definitions

Data on sex, age, presence of indwelling catheter, documentation of date and time of collection, and processing time (i.e., time from specimen collection to the start of incubation) were collected. The processing was categorized as less than 4 h, 4–24 h, or ≥ 24 h. Urine cultures with growth of an organism  ≥ 100,000 CFU/mL were considered positive culture results for the purpose of this analysis.

The primary outcome was culture positivity, which is the proportion of cultures with significant bacterial growth.

### Statistical analysis

Descriptive statistics were employed to summarize the demographic data and growth rates. Categorical variables were presented as frequencies (%) and continuous variables were represented as median and interquartile range. We used Chi-square to study the associations between categorical variables and having a positive urine culture. Continuous variables were evaluated with either Kruskal-Wallis test or Mann-Whitney test. To identify independent predictors of significant bacterial growth, we used multivariable logistic regression. We repeated the regression, stratifying by processing time. SPSS software was used for all statistical analyses (IBM SPSS Statistics, ver. 28, IBM corp., Armonk, NY, USA, 2021). This study was approved by the Institutional Review Board; the requirement for informed consent was waived.

## Results

### Patient and urine culture characteristics

During the study period, a total of 12,660 urine cultures were processed, of which 9117 samples were ordered before the intervention and 3543 tests were ordered after the intervention. The characteristics of patients and urine cultures are summarized in Table 1. When comparing the pre-intervention and post-intervention periods, there was a higher proportion of male patients in the post-intervention phase (52.1% vs. 48.5%, *p* < 0.001). The median age of patients was lower in the post-intervention phase (80 years vs. 81 years, *p* < 0.001). No significant difference was observed in the prevalence of indwelling urinary catheters.

**Table 1:** Patients and urine cultures characteristics during the pre- and postintervention periods

Variables		2020 *n* = 4683	2021 *n* = 4434	2022 *n* = 3543	Total *n* = 12,660	*P*
Patient characteristics	Gender, male	2297^a^ (49.0)	2127^a^ (48.0%)	1845^b^ (52.1%)	6269 (49.5%)	0.001
	Age, median (IQR)	81 (71–88)	82 (70–88)	80 (70–87)		< 0.001
	Indwelling catheter	2762^a^ (59.0)	2669^a^ (60.2)	1868^a^ (58.1)	7299 (59.2%)	0.182
Collection time	Full documentation (date + time)	1852^a^ (39.5)	1442^b^ (32.5)	1536^c^ (43.4)	4830 (38.2)	< 0.001
	Day shift	717^a^ (38.7)	519^a^ (36.0)	696^b^ (45.3)	1932 (40.0)	< 0.001
	Evening shift	721a (38.9)	576a (39.9	514b (33.5)	1811 (37.5)	
	Night shift	414^a^ (22.4)	347^a^ (24.1)	326^a^ (21.2)	1087 (22.5)	
	Processing time, hours median (IQR)	14 (5, 20)	14 (6, 19)	11 (4, 18)	13 (10, 18)	< 0.001
	Processing within 4 h	393^a^ (21.2)	236^b^ (16.4)	405^c^ (26.4)	1034 (21.4)	< 0.001
	Processing within 4–24 h	1244^a^ (67.2)	1073^b^ (74.4)	1047^a^ (68.2)	3364 (69.7)	
	more than 24 h	215^a^ (11.6)	133^b^ (9.2)	83^c^ (5.4)	431 (8.9)	
Culture results	Negative	2314^a^ (49.4)	2164^a^ (48.8)	1974^b^ (55.7)	6452 (51.0)	< 0.001
	Mixed growth	319^a^ (6.8)	395^b^ (8.9)	251^a^ (7.1)	965 (7.6)	
	Growth < 10^5^ CFU	671^a^ (14.3)	611^b^ (13.8)	440^b^ (12.4)	1722 (13.6)	
	Growth > 10^5^ CFU	1379^a^ (29.4)	1264^a^ (28.5)	878^b^ (24.8)	3521 (27.8)	

Note Data are presented as numbers (percentages) unless otherwise indicated

CFU colony forming units; IQR interquartile range

Each superscript letter denotes a distinct subset of year categories. Columns with the same superscript indicate no significant differences at the 0.05 significance level

Complete documentation of collection dates and time was recorded for 38.3% (4830 /12,660) of the specimens. The median processing time has decreased from 14 h (IQR 7–19) to 11 h (IQR 4–7) *p* < 0.001. During 2022, 26% of urine samples were processed within 4 h, compared to 21% and 16% during 2020 and 2021, respectively (*p* < 0.001).

### Culture positivity

As shown in Table 2, culture positivity was associated with several factors. This included sex, with a positivity rate of 31.0% (763/2458) among females compared with 23.4% (555/2371) among males (*p* < 0.001); The median age of patients with positive cultures was 83 years (IQR 74–89) compared with 80 years (IQR 69–87) among those with negative or non-significant growth. Samples obtained through indwelling urinary catheters exhibited a positivity rate of 31.2% (962 /3088), while midstream collection yielded a lower rate of 20.9% (332 / 1586). Culture positivity was higher with longer processing times: positivity was 21.3% (220/1034) when specimens were processed within 4 h, 28.4% (955/3364) when processed in 4–24 h, and 32.9% (137/417) when processed after 24 h (*p* < 0.0001).

**Table 2:** Characteristics of positive versus negative urine cultures

Variable		Positive urine cultures (*N* = 1318)	Negative urine cultures (*N* = 3511)	*P*
Age, median (IQR)		83 (74–89)	80 (69–87)	< 0.001
Sex, Female		763 (57.9%)	1816 (51.7%)	< 0.001
Indwelling urinary catheter		962 (73%)	2126 (60.6%)	< 0.001
Shift	Day	450 (34.1%)	1482 (42.2%)	< 0.001
	Evening	557 (42.3%)	1254 (35.7%)	
	Night	311 (22.1%)	772 (23.6%)	
Processing time	< 4 h	220 (16.7%)	814 (23.2%)	< 0.001
	4–24 h	995 (72.5%)	2409 (68.6%)	
	>24 h	143 (10.9%)	280 (8.0%)	

*Note* Data are presented as numbers (percentages) unless otherwise indicated

IQR interquartile range

**Fig. 1:**
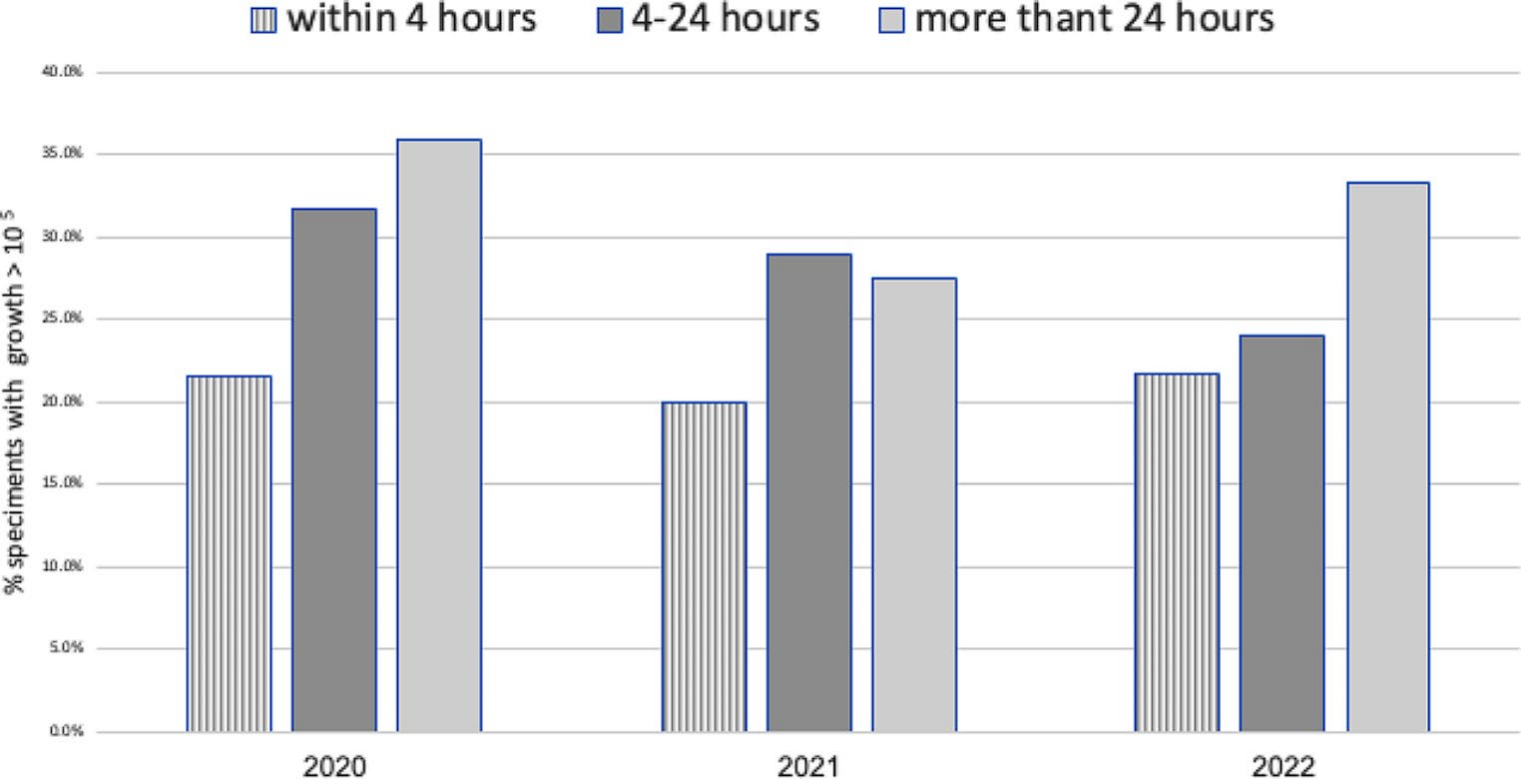
Positive urine culture ratio in correlation with the duration between collection and transport time to the microbiology laboratory

Culture positivity decreased from 28.9% pre-intervention to 23.8% post-intervention (*p* < 0.001). Among specimens processed within 4–24 h, positivity decreased significantly from 30.4% (704//2317) to 24.0%;(251/1047) while no significant changes were observed for specimens processed < 4 or ≥ 24 h (Fig. 1). A multivariate analysis of fully documented samples revealed multiple variables linked to increase risk of positive results, including age, gender, and presence of indwelling catheter (Table 3). Collection specimens during evening (OR 1.37;95% CI 1.15–1.63; *p* < 0.001) or night shifts (OR 1.25, 95%CI 1.03–1.52; *p* = 0.03) were associated with increased positivity compared to day shift. Cultures processed after 24 h were independently associated with increased risk. (OR 1.69, 95%CI 1.30–2.20); *p* < 0.0001) Further stratified analysis, by processing timing, revealed that the intervention significantly reduced positive culture only for specimens processed between 4 and 24 h (OR 0.80, 95% CI 0.67–0.94; *p* = 0.008).

**Table 3:** Multivariable logistic regression for predictors of significant bacterial growth in urine cultures

	Variable	OR (95% CI)	*P*
All time frames	Study phase		
	Pre-intervention	Reference	
	Post-intervention	0.88 (0.77, 1.02)	0.09
	Age, median (IQR)	1.02 (1.01, 1.02)	< 0.001
	Female	1.37 (1.20, 1.57)	< 0.001
	Indwelling urinary catheter	1.41 (1.21, 1.64)	< 0.001
	Shift		
	Day	Reference	
	Evening	1.37 (1.15, 1.63)	< 0.001
	Night	1.25 (1.03, 1.52)	0.03
	Processing time		
	< 4 h	Reference	
	4–24 h	1.21 (0.99, 1.48)	0.061
	>24 h	1.69 (1.30, 2.20)	< 0.001
Analysis per time			
< 4 h	Intervention	1.12 (0.82, 1.54)	0.47
	Age	1.02 (1.01, 1.03)	0.001
	Female	1.57 (1.15, 2.14)	0.005
	Indwelling urinary catheter	1.45 (1.92, 2.06)	0.041
4–24 h	Intervention	0.80(0.67, 0.94)	0.008
	Age	1.02 (1.01, 1.02)	< 0.001
	Female	1.42 (1.21, 1.66)	< 0.001
	Indwelling urinary catheter	1.40 (1.17, 1.67)	< 0.001
≥ 24 h	Intervention	1.17 (0.71, 1.93)	0.54
	Age	1.02 (0.99, 1.03)	0.07
	Female	0.80 (0.53, 1.22)	0.30
	Indwelling urinary catheter	1.47 (0.91, 2.38)	0.11

## Discussion

The objective of this study was to explore urine collection practices and evaluate the impact of utilizing boric acid tubes on quantitative urinary bacterial cultures among hospitalized patients. We found that under standard clinical conditions, the majority of urine cultures were not processed within a 4-hour timeframe. This delay in processing was associated with higher culture positivity. Additionally, increased positivity was observed for cultures collected in the afternoon or night compared to day shifts. The introduction of boric acid transport tubes led to a notable reduction in positivity. Further analysis demonstrated that this intervention had the greatest impact on specimens processed within 4–24 h post-collection.

Delays in specimen transport often challenge laboratories in maintaining the stability of urine samples submitted for culture. Research conducted in the 1970s investigated the impact of transport delays on semiquantitative urine culture results [6, 7]. In line with the findings of these studies, it is recommended to maintain a maximum four-hour interval between urine collection and processing [5]. However, in a survey of US laboratories, only a small number of microbiology laboratories enforced cutoff rule of 4 h for limiting transport time of urine [15]. Notably, in that survey, 90% of the specimens in the surveyed laboratories were received within 9 h. Refrigeration of urine specimens has been shown to limit the overgrowth of organisms [7]. However, temperature-controlled transport is rarely feasible in routine clinical and laboratory settings. Only 35% of laboratories surveyed refrigerated the specimens before or during processing [15]. In the current study, processing time appeared to have a significant impact on culture positivity. We observed an increase in positivity following four hours of sample storage at room temperature, with a more substantial increase noted after 24 h.

Boric acid effectively inhibits the replication of bacteria in urine samples, maintaining the initial quantity of bacteria present at the time of collection. Boric acid preservation has been shown to be comparable to 24 h of refrigeration [16]. Given that the transport duration could exceed the four-hour window, the employment of urine preservatives may be an effective strategy to minimize overgrowth. In one study, boric acid was effective in maintaining the stability of urine specimens for up to 48 h at room temperature [17]. In contrast, Daley et al. found a notable increase in significant bacterial growth when cultures were stored in boric acid tubes for more than 24 h compared to those analyzed immediately. This trend was more evident at 72 h [16]. Likewise, subsequent to the adoption of boric acid transport tubes, our investigation demonstrated a marked reduction in positive cultures only for specimens processed within the 4–24 h window. This suggests a time-dependent effect of boric acid on inhibiting growth, with its potency diminishing after 24 h. Consequently, certain bacterial species may begin to proliferate despite its presence. Therefore, for accurate culture results, urine sample in boric acid tubes should be analyzed within 24 h.

Our investigation has identified notable concerns and opportunities for improvement: A significant portion of collected samples lacked documented collection times. In addition, the majority of specimens were not transported within the recommended 4-hour window. Various factors can contribute to delays in transporting and processing microbiology specimens, potentially jeopardizing patient safety. One such factor is unfamiliarity with the guidelines for timely urine specimen transmission. Additionally, the mode of transportation, whether through manual porters or pneumatic tube systems, can impact the time it takes for samples to reach the laboratory. When pneumatic tube systems are absent, reliance on porter availability becomes a bottleneck, resulting in delays in sample delivery to the lab. Moreover, a significant number of laboratories do not offer 24-hour services. Findings from a survey conducted across four European countries indicated that most laboratories are closed overnight, and merely around 40% maintained services during weekends [18]. Consequently, cultures obtained outside of regular operating hours are often stored for extended periods in clinical wards, typically at room temperature. Our investigation has revealed a clear link between the timing of urine culture collection and the subsequent ratio of positive results. Notably, cultures collected during daytime shifts exhibit lower positivity compared to those acquired during evening and night shifts. These observations indicate that operational hours of the laboratory and the allocation of daily human resources exert a more pronounced influence than the mere growth kinetics of the microorganisms themselves.

The before-after design does not account for other changes in practices that may influence urine culture positivity. During the intervention period, we initiated educational activities aimed at promoting appropriate indications for urine cultures. The effect of this intervention is reflected by the decreased number of cultures collected in 2022, as shown in Table 1. Reducing unnecessary cultures is generally expected to increase urine culture positivity, as noted in a previous study [14]. Therefore, the reduction in positivity observed in the current study is unlikely to be explained by a decrease in urine culture orders.

Our study was subject to various limitations. The research was conducted in a single acute-care hospital, which may limit the generalizability of the findings to other healthcare facilities with different patient populations, practices, and resources. Furthermore, the data collected included minimal information on patient characteristics. The absence of comprehensive patient details, including medical histories and clinical data, potentially overlooks crucial factors that may influence the culture results. The absence of a control group is another potential limitation, making it difficult to ascertain the impact of the laboratory intervention versus other coexisting variables. Nevertheless, it is noteworthy that the reduction in positivity was specifically observed at the 4–24 h processing window, hinting at a plausible connection with the intervention undertaken. Complete documentation was available for only a subset of specimens. Consequently, our ability to assess the impact of processing time was limited to a relatively small portion of the cultures.

In summary, our study underscores the importance for healthcare facilities to re-evaluate and optimize their urine culture transport and storage protocols. We identified substantial delays in transportation, prompting the adoption of boric acid tubes, which resulted in a marked reduction in positivity rates, particularly within the critical 4–24 h processing window. This intervention shows significant potential in enhancing the precision of UTI diagnosis accuracy, thereby contributing to better antibiotic stewardship,

## Data Availability

No datasets were generated or analysed during the current study.
